# The Dental Digest

**Published:** 1895-02

**Authors:** 


### THE DENTAL DIGEST.

This new journal, as its title-page indicates, is to be "A Monthly Summary of Dental Science devoted to the Progress of Dentistry." It will be the official organ of the "Dental Protective Association of the United States" and also the "Dental Protective Supply Company" of Chicago.

It is presumed it has editors, as its sixty-four pages indicate labor in preparation, but no names are announced as conductors. The January issue, Vol. I., No. 1, is as a whole an interesting number and well prepared. The illustrations leave much to be desired, especially those of the American Dental Society of Europe. They are certainly not self-explaining.

The "Digests," prepared, it is presumed, by Dr. Crouse, make a very readable *résumé* of various society reports, but it is doubtful whether readers will gain much from a paragraph taken here and there as a substitute for an entire paper.

We wish our contemporary success in its chosen field.

